# Apnoeic oxygenation with high-flow nasal oxygen in adult patients undergoing endoscopic pharyngolaryngeal surgery: a clinical review

**DOI:** 10.1097/EA9.0000000000000114

**Published:** 2026-06-03

**Authors:** Sophie Harris, Alistair F. McNarry, Patrick A. Ward

**Affiliations:** From the Western General Hospital, Crewe Road South, NHS Lothian, Edinburgh (SH, AFM), St. John's Hospital, Howden West Road, NHS Lothian, Livingston, UK (AFM, PAW)

## Abstract

Endoscopic pharyngolaryngeal surgery requires access to the base of tongue, pharynx, larynx, trachea and/or upper oesophagus using a rigid or flexible endoscope for diagnostic or therapeutic surgical management of benign or malignant disease. The anaesthetist must deliver a safe and effective shared airway management strategy. This strategy is aimed at optimising the surgical view and access to pathology, whilst avoiding airway obstruction, maintaining oxygenation, facilitating carbon dioxide clearance, and minimising the risk of airway fire.

Various strategies exist to achieve this depending on the location, size and nature of the pathology, patient anatomy and comorbidities, and operating team experience. One such approach is apnoeic oxygenation using high-flow nasal oxygen. Over the last decade, this has become a widely adopted ‘tubeless’ anaesthetic technique; nevertheless, it cannot be used in all patients, for all pathologies, by all operating teams. Though our understanding of the physiological principles continues to evolve, it is clear that to deliver this technique optimally, there are several important considerations that the surgical, nursing and anaesthetic teams must be aware of. A collaborative team-based approach to case selection and planning is essential. This must be paired with an appreciation of the limitations of the technique and the need to have an immediately deliverable rescue oxygenation strategy in place.

This article provides an overview of the likely physiological mechanisms, the benefits, the limitations, the alternatives and the important considerations involved in the safe and effective use of apnoeic oxygenation with high-flow nasal oxygen to facilitate endoscopic pharyngolaryngeal surgery.

## Introduction

Effective patient oxygenation during surgery is a fundamental principle of safe anaesthesia. In certain circumstances, conventional oxygenation and lung ventilation can impede surgical access. An example of this is endoscopic pharyngolaryngeal surgery, which includes procedures such as microlaryngoscopy, panendoscopy, tissue biopsy, dilatation of stenoses and laser treatment of pathology/suspected pathology of the base of tongue, pharynx, larynx, trachea and upper oesophagus. Most commonly, the surgeon performs direct laryngoscopy, using a rigid suspension laryngoscope to evaluate the upper airway and/or oesophagus, focusing on the anatomical area of concern, prior to tissue biopsy and/or management of the pathology. Various oxygenation (and lung ventilation) strategies have been described for this scenario, and can be broadly classified as closed or open systems. Their selection is based upon the size, location and nature of the pathology (including bleeding risk), anticipated operation duration, patient comorbidities, anatomical differences, and the preference of the surgeon, anaesthetist and institution. Closed systems refer to the use of a cuffed tracheal tube (standard, microlaryngeal or laser-resistant), permitting conventional mechanical ventilation of patients’ lungs, and allowing precise control and monitoring of respiratory parameters (including end-tidal carbon dioxide). Open systems maintain a direct connection between patients’ lower airways and the surrounding atmosphere, and include high-pressure jet ventilation (low, high or dual frequency delivered via supra-, infra- or trans-tracheal routes), spontaneously breathing insufflation and apnoeic oxygenation techniques, where accurate control and monitoring of respiratory parameters may be more challenging (or impossible). Transition between closed and open systems in the same patient during the course of a surgical procedure may be required.

Apnoeic oxygenation describes oxygenation achieved in the (intentional) absence of spontaneous respiration/respiratory effort or artificial/mechanical lung ventilation.^1^ The concept is not new, having been originally described in 1667 by Hook during an experiment involving inflation of canine lungs.^2^ Apnoeic oxygenation using low gas flows was explored further during the 1940s and 1950s and was shown to be a relatively effective means of oxygenating patients. However, interest declined due to the significant issue of carbon dioxide (CO_2_) accumulation and the negative sequelae from the resultant respiratory acidosis. More recently, a number of specific indications for apnoeic oxygenation with *low* gas flows have emerged, including during brain stem death testing, one lung ventilation (oxygenation of the dependent, non-ventilated lung) and interventional radiological procedures where elimination of respiratory motion artefact is desirable (e.g. ablation of liver lesions). Patel and Nouraei's study in 2015^3^ renewed researchers’ and clinicians’ interest in apnoeic oxygenation and specifically for use during ‘tubeless’ airway surgery. They showed it to be an effective oxygenation strategy facilitating optimal surgical views, but also their findings suggested that the delivery of *high* gas flows may attenuate CO_2_ accumulation; prompting the technique to be termed ‘transnasal humidified rapid-insufflation ventilatory exchange’ (THRIVE). This technique offered several other potential advantages over existing oxygenation methods:

(1) Low pressure system, reducing the risk of barotrauma associated with jet ventilation
(2) Clear visualisation of the glottis, which a tracheal tube/jet catheter might obscure
(3) Reduction in the number of interruptions during surgery, common with intermittent apnoea/ventilation

Several physiological mechanisms were suggested to explain patient oxygenation and CO_2_ clearance during apnoeic oxygenation with high-flow nasal oxygen (HFNO-ap). However, subsequent studies have disproven the ventilatory component of THRIVE, having shown that the rate of CO_2_ accumulation is *independent* of gas flow.^4,5^ Despite an incomplete understanding of the underlying mechanisms, HFNO-ap has become widely adopted as a means of oxygenating patients during endoscopic pharyngolaryngeal surgery. Further research is required to clarify the processes that influence gas exchange. The technique can still be used in clinical practice provided meticulous case selection is undertaken; HFNO-ap cannot be delivered safely and effectively in all patients, for all endoscopic surgical procedures, by all operating teams. Patient factors, equipment factors and team training/experience must all be considered when determining if HFNO-ap is the optimal oxygenation strategy for an individual patient.^6^ A collaborative team-based approach is crucial throughout all stages of patient assessment, preparation and perioperative care to ensure that technique limitations are recognised and addressed, and that a patient-specific rescue oxygenation strategy is immediately deliverable.^7^

## History of apnoeic oxygenation and underlying principles

Oxygenation of human subjects (rather than animals) in the absence of spontaneous respiration or mechanical ventilation was demonstrated by Comroe and Dripps in 1946^8^ and by Enghoff *et al*.^9^ The latter demonstrated that after approximately 5 min of apnoea in anaesthetised patients, the oxygen content of arterial blood did not fall from baseline when 100% oxygen was delivered continuously to the lungs via a tracheal tube. They concluded that the success of apnoeic oxygenation was based upon preoxygenation, maintenance of a patent airway, delivery of a high fractional inspired oxygen concentration during apnoea and an adequate circulation – principles that still underpin effective delivery of this technique today.

### Aventilatory mass flow

One of the first suggested physiological theories underlying HFNO-ap was termed ‘diffusion respiration’. During apnoea, oxygen consumption by the body's cells continues, such that uptake of oxygen from the alveoli (250 ml min^−1^) exceeds CO_2_ returning from the bloodstream (8–20 ml min^−1^).^10^ This creates a sub-atmospheric pressure gradient within the alveoli promoting *mass* movement of gas from the upper airway to the alveoli (now often called ‘aventilatory mass flow’). If high concentrations of oxygen are continuously delivered to the pharynx and upper airways, bulk flow of oxygen continues to the alveoli, whilst a preferential diffusion gradient persists. The magnitude of the initial pressure gradient for oxygen transfer at the onset of apnoea will be reduced if effective preoxygenation (de-nitrogenation) is not undertaken. Over time, once CO_2_ buffering and storage capacity have been exceeded, CO_2_ accumulation within the alveoli leads to loss of the pressure gradient and curtails this process of bulk flow^1^ – as well as leading to hypercarbia, acidaemia and cardiovascular complications that limit the technique as a whole.

In addition to aventilatory mass flow, other physiological theories have been suggested that may contribute towards the success of HFNO-ap, and are described briefly below.^1^

### Dead-space flushing

Möller *et al.* ^11^ used a radioactive tracer gas and scintigraphy in healthy volunteers to demonstrate that HFNO improved gas exchange by increased clearance of anatomical dead space gases in a time and rate dependent manner, although its contribution to improved oxygenation during *apnoea* is likely to be minimal compared with spontaneously breathing patients.

### Positive airway pressure

Studies have demonstrated benefit from generation of positive airway pressure with HFNO in spontaneously breathing patients. These include improved airway patency,^12^ increased end-expiratory lung volume (increased functional residual capacity), reduced work of breathing, improved end-expiratory lung volume impedance, higher arterial oxygen partial pressure/fractional inspired oxygen ratios,^13^ cumulative and linear increase in end-expiratory lung impedance with increasing gas flows and a reduction in respiratory rate.^14^ The degree of positive airway pressure generated within the nasopharynx varies according to whether subjects are breathing with their mouths open (~0.5 cmH_2_O per 10 l min^−1^) or closed (~1 cmH_2_O per 10 l min^−1^).^15^ During apnoea, positive airway pressure generation is likely to be negligible however, especially if the mouth is open to permit surgical access.

### Warmed, humidified gas delivery

The benefits of delivering HFNO heated to 37 °C and humidified to 44 mg H_2_O l^−1^ are greater in the awake spontaneously breathing patient than in the anaesthetised, apnoeic patient (improved tolerance of high gas flows, reduced work of breathing); nevertheless, humidified oxygen minimises airway constriction, improves mucociliary clearance (reducing mucus plug formation) and reduces atelectasis.^16^

### Extension of turbulent flow into distal airways

Turbulent gas flow (as opposed to laminar) is associated with improved gas exchange. With higher rates of gas delivery, turbulent gas flow that occurs in the larger upper airways *may* extend into smaller distal airways, promoting gas exchange and CO_2_ clearance during apnoea. Though largely restricted to animal studies (and thus, considered speculative), one study with 11 patients,^17^ that involved continuous insufflation of 45 l min^−1^ oxygen via endobronchial catheters, demonstrated clinically acceptable partial pressures of oxygen and CO_2_ within arterial blood during 20 to 30-minute procedures.

### Cardiogenic mixing

Cardiac oscillations may promote gas exchange and CO_2_ clearance. The pumping action of the heart against adjacent lung tissue may directly promote gas flow within the lung, and changes in intrathoracic pressure from variation in cardiac volume throughout the cardiac cycle may indirectly promote gas exchange within the lung. Hermez *et al.* ^18^ demonstrated enhanced CO_2_ clearance during apnoea in three different laboratory models when oxygen flow rates were increased from 20 to 70 l min^−1^, attributing this finding to the interaction of highly turbulent supraglottic flow vortices (created by HFNO) and cardiogenic oscillations. Turbulent eddies were visualised within the pharynx caused by HFNO being entrained into the trachea during cardiogenic inspiration.

THRIVE was described by Patel and Nouraei in 2015 as a novel technique using HFNO to prolong the ‘safe apnoea time’ in 25 patients with predicted difficult airway management/increased propensity for oxygen desaturation undergoing hypopharyngeal and laryngotracheal surgery.^3^ Humidified oxygen at high gas flows (up to 70 l min^−1^) was delivered to patients via nasal cannulae, starting from preoxygenation of the awake spontaneously breathing patient positioned in a 40° head-elevated position, during induction of general anaesthesia (with administration of neuromuscular blockade), throughout surgery in the apnoeic patient, until a definitive airway was required or spontaneous respiration was restored. ‘Apnoea time’ was defined as the time between neuromuscular blockade and commencement of an alternative oxygenation strategy or resumption of spontaneous ventilation. The median apnoea time was 14 min and no patients experienced oxygen desaturation <90%. THRIVE was utilised as the sole means of oxygenation in two patients (apnoea times of 32 and 65 min). Distinct from previous studies involving apnoeic oxygenation with low gas flows, Patel and Nouraei suggested aventilatory mass flow was not the only mechanism of oxygenation during apnoea with high gas flows, attributing their findings to the generation of positive airway pressure and reduced pulmonary shunt fraction.^19–21^

Patel and Nouraei also suggested that the high oxygen flows also contributed towards enhanced clearance of CO_2_ (slower rate of accumulation) than previously demonstrated during ‘classical’ apnoeic oxygenation with low gas flows,^22–24^ proposing that this was related to improved gaseous mixing and dead space flushing. This finding challenged the major limitation of apnoeic oxygenation up to that point – build-up of CO_2_, the associated acidosis and its negative sequelae.

THRIVE offered the potential advantages of a ‘tubeless’ surgical field, unimpeded glottic views, no prior instrumentation, an effective means of oxygenation for short surgical procedures, delivered by a low-pressure system, that potentially also attenuated CO_2_ accumulation. Several studies and case series were published^25–28^ in this specific area of practice, reflecting the widespread adoption of THRIVE for pharyngolaryngeal surgery (among other clinical applications of HFNO-ap).

Recent studies have challenged Patel and Nouraei's original observations regarding enhanced CO_2_ clearance with THRIVE. Two studies in children^29,30^ demonstrated no difference in the rate of CO_2_ accumulation with varying oxygen flow rates. Subsequently, these findings have been replicated in adults,^4^ prompting an editorial that recommended the suggested ventilatory exchange (VE) component of THRIVE should be dispensed with.^31^ A number of assumptions were made in the original THRIVE study that might explain these different findings.

Patel and Nouraei inferred that the rate of rise of CO_2_ during HFNO-ap was attenuated by making comparison with historical low-flow apnoeic oxygenation studies rather than contemporaneous control subjects. Heterogeneity within the various study populations, trial protocols and drug regimens (including administration of large doses of suxamethonium – associated with increased CO_2_ production from muscle fasciculations) may account for their overestimate of CO_2_ clearance. Additionally, the rate of CO_2_ accumulation during apnoea is non-linear, meaning that the end-point selected by different studies may introduce discrepancies. Finally, end tidal CO_2_ levels were recorded in 80% of the patients in their study rather than arterial blood levels, which have been shown to significantly underestimate arterial CO_2_ levels after an extended period of apnoea.^25^ Given these limitations and the findings of other studies^5^ that confirm the rate of CO_2_ accumulation is independent of oxygen flow rates, ‘THRIVE’ may not be the most appropriate or accurate abbreviation.

## Benefits of HFNO-ap in pharyngolaryngeal surgery

### Unobstructed surgical field

The ‘shared airway’ refers to the clinical situation where the anaesthetist and surgeon must share the same anatomical space, working cooperatively to safely facilitate adequate airway patency, oxygenation and CO_2_ clearance whilst permitting surgery to the upper aerodigestive tract. Depending upon the location, size and nature of the pathology, patient comorbidities and anatomical differences, and the operating team preferences, a number of different oxygenation strategies are available. The most significant advantage that HFNO-ap offers is the unobstructed (‘tubeless’) surgical field, where the surgeon's view (of the airway and any pathology) and operative space (access to pathology) is not impaired by the presence of a tracheal tube or jet ventilation catheter. Provided airway patency is maintained by appropriate positioning of the surgical laryngoscope, the surgeon can visualise the entire upper airway whilst oxygen continues to flow from the nares to alveoli. Their field of view is not restricted by the necessity to align the laryngoscope with the glottic opening, as is required for the safe delivery of high-pressure supraglottic jet ventilation (to avoid dissection of tissue planes/subcutaneous emphysema). In comparison, the presence of a microlaryngeal tube, jet ventilation catheter or even ultra-thin Tritube (Ventinova Medical, Eindhoven, the Netherlands) can obstruct the surgeon's view of the glottis,^32^ and potentially obscure access to pathology – particularly for lesions of the posterior third of the vocal cords.

### Reduced airway instrumentation

Complications can occur during any airway management technique and HFNO-ap must never be regarded as a fail-safe technique; however, it may confer some benefits. The avoidance of instrumentation for airway management not only allows better visualisation of the entire glottis, but avoids any inadvertent trauma/bleeding from the tracheal intubation process. This can be especially beneficial for subtle lesions, friable tissues or highly vascularised mucosa (e.g. pregnant patients with engorged tissues requiring balloon dilatation of subglottic stenosis).^33^ The fourth National Audit Project (NAP4) of airway complications in the UK also showed that repeated airway instrumentation was associated with an increased incidence of adverse events.^34^

Following surgery, as the patient transitions from apnoea to spontaneous respiration, HFNO may continue without the insertion of a supraglottic airway or tracheal tube. However, in this situation, a patent airway must still be maintained with simple manoeuvres, e.g. jaw thrust, until resumption of adequate spontaneous respiration and return of protective airway reflexes. The decision to use HFNO only at emergence must be based upon the individual patient, particular pathology, specific surgical procedure, efficacy of perioperative oxygenation/requirement for rescue interventions, pulmonary aspiration risk etc.

### Effective method of oxygenation

Patel and Nouraei demonstrated that THRIVE was an effective means of oxygenating patients undergoing upper airway surgery.^3^ Since their 2015 study, HFNO-ap has consistently been shown to be a safe and effective oxygenation technique in a range of different patient populations undergoing short endoscopic upper airway diagnostic and/or therapeutic procedures,^26,30,33^ with 95% of patients in one case series tolerating HFNO-ap for 20 min without oxygen desaturation.^26^

In order for the duration of apnoeic oxygenation to be maximised, effective preoxygenation must be undertaken (this avoids the preferential pressure gradient for oxygen transfer being reduced by the presence of nitrogen in the alveoli). Airway patency must also be maintained throughout every stage of anaesthesia and surgery. High gas flows and a high fractional inspired concentration of oxygen should be used as the safe apnoea time is reduced at lower flows,^35^ and lower inspired concentrations.^36^ Optimal preoxygenation and subsequent apnoeic oxygenation can be achieved by placing the patient in a ‘ramped’ position, though the ideal 40–45° incline may not always be feasible for the entire surgical procedure. Compromise may be required to allow for patients’ body habitus and positioning of the suspension laryngoscope to maintain optimal surgical views/accessibility. Schutzer-Weissmann *et al*.'s trial of HFNO-ap (70 l min^−1^) in patients with a raised body mass index demonstrated the importance of ramped positioning – even though the control group received only 15 l min^−1^ oxygen delivered via a tightly fitted facemask, a significant proportion of *both* groups underwent successful apnoeic oxygenation for 18 minutes; only 5/41 (12%) patients in the HFNO-ap group and 14/39 (36%) patients in the facemask group experienced oxygen desaturation, SpO_2_ ≤ 92%. Overall, the risk of oxygen desaturation was significantly lower in the HFNO-ap group (hazard ratio = 0.27; 95% confidence interval 0.11 to 0.65; *P* = 0.007).^37^ This trial also highlights the fundamental principle of successful apnoeic oxygenation – maintenance of a patent airway, even if adjuncts are required.

### Low-pressure system

Whilst gas flows are high with HFNO (generally 50–70 l min^−1^ in adults; exceeding peak inspiratory flow^38^), the pressure transmitted to patients’ airways is low, such that the risk of iatrogenic barotrauma is likely to be significantly lower than when using a high-pressure oxygen source. This is especially the case in low frequency manual jet ventilation, where there is no pressure monitoring facility or pause pressure function, as with automated high-frequency devices.^39^ Concerns over gastric insufflation, distension and associated sequelae have largely been disproven by ultrasound studies assessing the cross-sectional area of the gastric antrum, showing no change with HFNO administration.^40^ Measured pressures are in the range of 0.5-1 cmH_2_O per 10 l min^−1^,^14,19^ which is well below the accepted threshold required to cause gastric insufflation (15–20 cmH_2_O).^41,42^

**Fig. 1:**
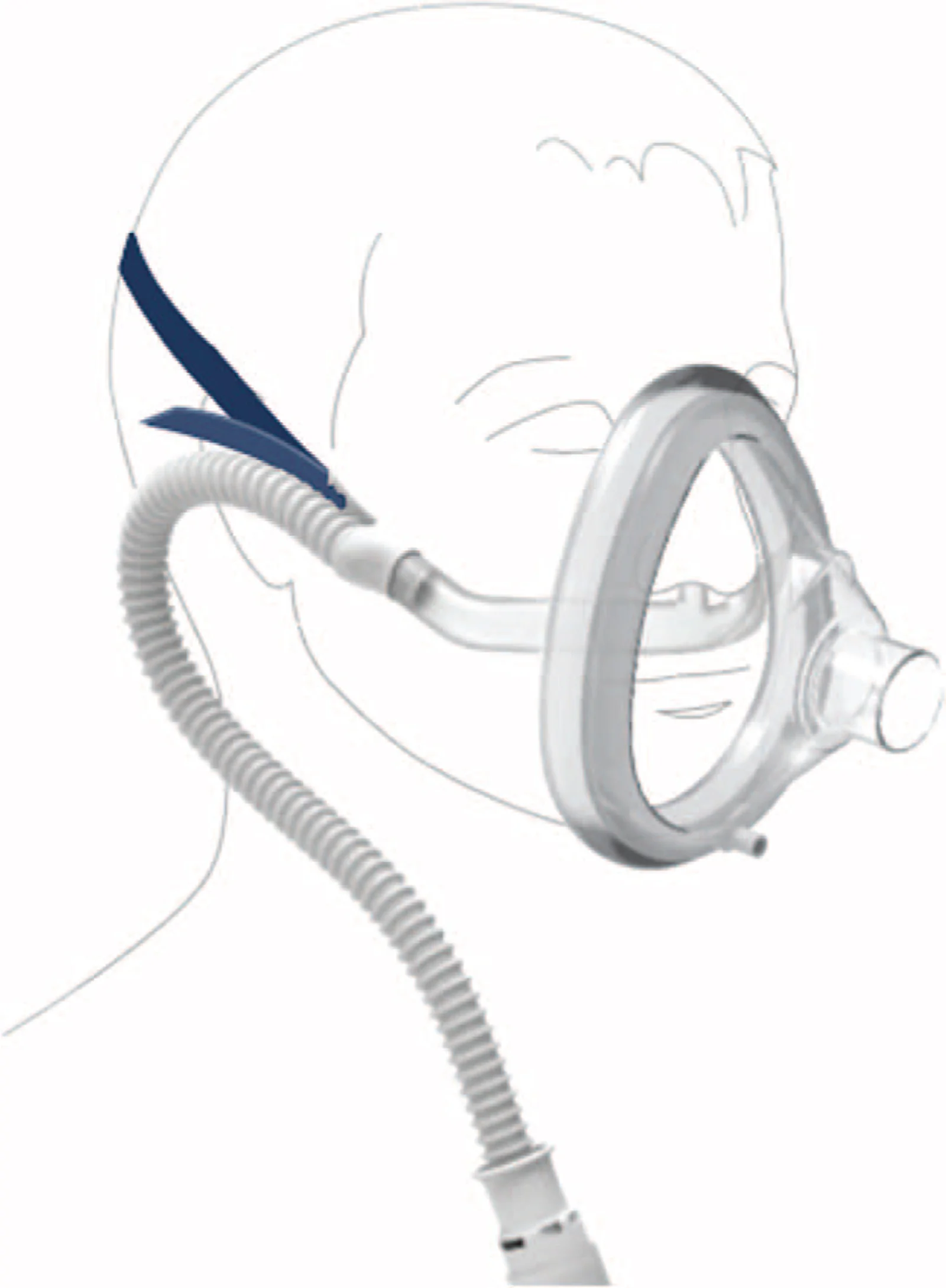
Optiflow Switch device with facemask placed directly over (and deliberately compressing) the oxygen delivery interface. Image courtesy of Fisher & Paykel Healthcare Limited (© 2021 Fisher & Paykel Healthcare Limited).

A pressure-sensing flow-diversion adaptation of the oxygen delivery system has recently been developed (e.g. Optiflow Switch, Fisher & Paykel Healthcare, Auckland, New Zealand), where the delivery arm of the cannula is compressible. When compressed, the HFNO is audibly diverted to the surrounding environment, whilst enabling a facemask seal (and lung ventilation) to be achieved with the cannula still *in-situ* (Fig. 1). This reduces the risk of barotrauma and facilitates rapid rescue oxygenation, if required. This ability to *immediately* convert from HFNO-ap to facemask ventilation may increase the number of patients in whom the technique is considered suitable.^43^

### Improved operating theatre productivity

A study comparing patients undergoing tracheal intubation and positive pressure ventilation via a microlaryngeal tube versus HFNO-ap for pharyngolaryngeal surgical procedures demonstrated reduced overall time in the operating theatre and reduced time in the post-anaesthesia care unit in the HFNO-ap group, with the potential to enable increased numbers of patients to undergo similar procedures on a single operating list.^44^

### Patient comfort and tolerability

In awake patients (i.e. during preoxygenation, at emergence and postoperatively), HFNO has been shown to be comfortable and well-tolerated, especially as the warmed, humidified gas is delivered via wide-bore soft nasal cannulae.^16^ Although the evidence for improved tolerability of HFNO is largely extrapolated from critical care (and prolonged) use,^45^ benefits have been demonstrated over facemasks (conventional anaesthetic facemask or non-rebreathe mask) for preoxygenation and following tracheal extubation,^46,47^ with patients reporting improved comfort, less claustrophobia and less anxiety.

## Limitations of HFNO-ap in pharyngolaryngeal surgery

### Maintenance of a patent airway

Airway patency is fundamental for the effective delivery of HFNO-ap.^4,7,8^ Whilst this is normally assured by appropriate positioning of the suspension laryngoscope, where any physiological or pathological factor (e.g. a large mobile tumour that obstructs the airway) make it impossible to maintain patency, an alternative oxygenation technique must be rapidly implemented.

### Carbon dioxide accumulation

The rate of CO_2_ accumulation is not attenuated by high gas flows, and whilst there is some clearance occurring during apnoea, the build-up of CO_2_ is still the most significant limitation of HFNO-ap. The effect of hypercapnia on the pulmonary, cardiovascular and cerebrovascular systems are of most concern – with the potential for serious morbidity/mortality in some cases. Review of an Australian online database of adverse anaesthesia-related incidents (webAIRS), identified two reports of complications relating to marked hypercarbia during HFNO use for endoscopic upper airway surgical procedures.^48^ In both cases, the extent of hypercarbia had been under appreciated, leading to cardiac arrest in one patient (from hypercarbia-induced exacerbation of pre-existing pulmonary hypertension) and prolonged duration of low conscious level post-procedure (requiring ventilatory support) in the other. Increased arterial CO_2_ leads to elevated pulmonary vascular resistance and right ventricular strain, and may potentially cause intracerebral steal phenomenon (where cerebral blood flow is paradoxically diverted away from poorly supplied vulnerable areas of the brain that are already maximally vasodilated, worsening ischaemia).

It is important to identify patients in whom CO_2_ accumulation may pose additional risk (e.g. pulmonary hypertension) and procedures that are likely to be prolonged.^7^ Such patients and procedures may be deemed unsuitable, and best conducted using an alternative oxygenation strategy, e.g. SponTaneous Respiration using IntraVEnous anaesthesia and Hi-flow nasal oxygen (STRIVE-Hi). STRIVE-Hi still provides a tubeless surgical field, but patients breathe spontaneously and CO_2_ accumulation is reduced,^49^ with some suggestion that procedural interruptions may also be fewer.^50^ Alternatively, HFNO-ap could be used for a particular phase of surgery, before switching to another means of oxygenation for the remainder of the procedure that facilitates restoration of normocarbia (e.g. positive pressure ventilation via a microlaryngeal tube).

In high-risk patients, arterial cannulation may facilitate frequent monitoring of perioperative CO_2_ levels. Otherwise, end-tidal CO_2_ monitoring can be used at the end of the procedure and potentially intermittently during surgery – though the benefit has to be weighed against the need to interrupt surgery, and the risk of underestimation of arterial CO_2_ levels must be fully appreciated. Transcutaneous CO_2_ monitoring may be available at some institutions. These devices allow continuous, non-invasive monitoring which can be a useful guide, although correlation of transcutaneous values and blood CO_2_ levels may not be entirely accurate. Sensors must be placed correctly (air bubbles trapped between the sensor and skin can affect reading accuracy) and there is a lag time. Pape *et al*.^51^ demonstrated “clinically acceptable” correlation between transcutaneous values and CO_2_ levels (albeit with underestimation at the start of the procedure and overestimation at the end); whereas, Schweizer *et al*.^52^ showed poor correlation between measurements and a lack of agreement between different transcutaneous monitoring devices.

### Unprotected airway

HFNO-ap can provide successful patient oxygenation but it does not provide airway protection by the very nature of being ‘tubeless’. This leaves patients’ airways vulnerable to pulmonary aspiration of either regurgitated gastric contents or blood from surgical activity. Haemostasis must be meticulous and can be more challenging to achieve in open oxygen systems such as this, given the relative contraindication to diathermy^53^ or LASER in such an oxygen-rich environment (see below). Premedication with prokinetic and proton pump inhibitor medications should be considered in patients with gastro-oesophageal reflux disease and/or other risk factors. Guidance on HFNO-ap use in patients taking Glucagon-like peptide-1 (GLP-1) agonists is yet to be clearly established.

### Oxygen desaturation

Even with effective preoxygenation, good patient positioning, the use of high gas flows and a high fractional inspired oxygen concentration, perioperative oxygen desaturation can still occur. Blood, secretions, tissue debris or surgical instruments can occlude the airway and studies have shown that certain patient comorbidities and particular patient groups are associated with increased risk of oxygen desaturation. Patients with pre-existing cardiovascular disease (especially hypertension)^54^ and respiratory disease (asthma, obstructive lung disease) may be more prone to hypoxaemic events – comorbidities that are particularly prevalent in adults undergoing endoscopic upper airway surgery for malignancy, due to their shared association with chronic smoking and alcohol consumption. Obesity (≥class III) and raised body mass index have consistently been associated with reduced duration of effective apnoeic oxygenation and increased requirement for rescue oxygenation interventions.^3,55,56^ This might be explained by increased airway resistance to gas flow, reduced lung compliance, decreased expiratory reserve volume and functional residual capacity, and increased intra-pulmonary shunt fraction. Under carefully controlled experimental conditions, HFNO-ap has been employed effectively in patients with a body mass index >40 kg m^−2^, but the patients were positioned at a 45° incline throughout, had no airway pathology and underwent no airway-related surgical intervention.^37^

Careful patient selection is key to the success of HFNO-ap,^6^ necessitating identification of patients at particularly increased risk of oxygen desaturation and selecting an alternative oxygenation strategy from the outset. In all patients deemed suitable, the multidisciplinary team must have an agreed, patient-specific, immediately deliverable rescue oxygenation strategy in place (whether facemask, supraglottic airway, jet ventilation or ventilation via a microlaryngeal tube).^7^ Additionally, once oxygen desaturation has occurred, HFNO-ap is not an effective re-oxygenation technique.

### Time limited nature

**Fig. 2:**
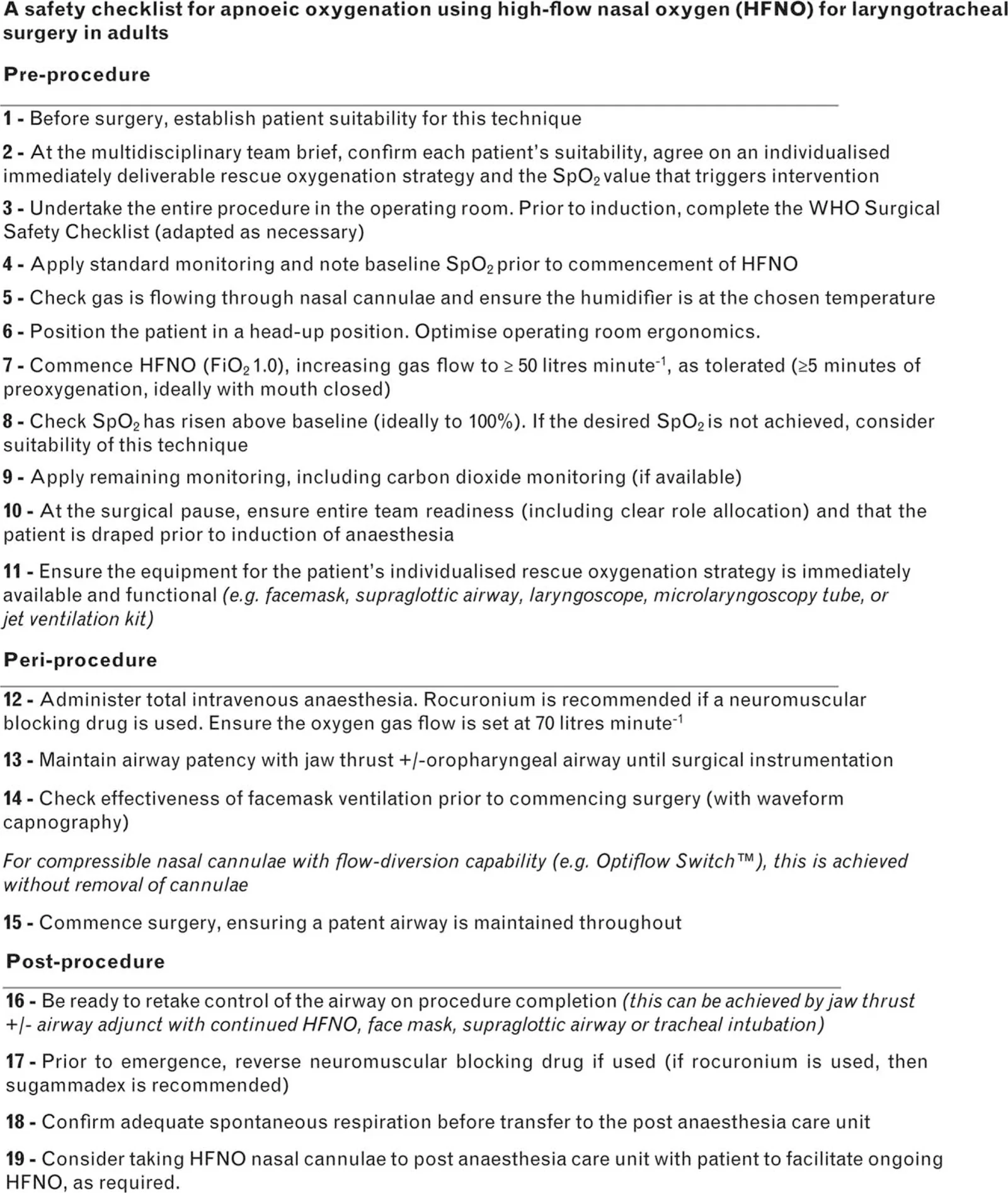
A safety checklist for apnoeic oxygenation using HFNO in adult patients undergoing laryngotracheal surgery, devised by an international Delphi consensus group (*European Journal of Anaesthesiology*).^7^

Aside from the issue and risks of CO_2_ accumulation in prolonged cases, the technique of HFNO-ap is ultimately curtailed by absorption atelectasis, leading to oxygen desaturation. The multidisciplinary team must be aware of this limitation and work together to minimise unnecessary delays to the start of surgery, maximising utility of the restricted time available. The team must function effectively together, know their equipment and be familiar with the operating theatre ergonomics. Regular team rehearsal and the use of a checklist (Fig. 2)^7^ can be invaluable in this regard.

### High resource requirements/specialist knowledge and equipment

Aside from positive pressure ventilation via a microlaryngeal tube, most oxygenation techniques in endoscopic upper airway surgery require specialist equipment requiring specialist knowledge for safe and effective use. In addition to the costs associated with device acquisition, maintenance and consumable items, most of these devices require high volumes of oxygen, which may limit their use in resource-limited healthcare systems. HFNO-ap is not the most expensive or specialised, when compared with alternatives such as dual frequency jet ventilation, modern cuirass ventilation^57^ or the tritube, that requires a specialist flow-controlled ventilator.^58^ Nevertheless, despite its apparent simplicity, HFNO-ap remains an *advanced* airway management technique, only to be used in carefully selected patients by teams trained in its delivery and familiar with the equipment.^7^ For example, the flow-diversion adaptation of Optiflow Switch can be unintentionally compressed by the surgeon's laryngoscope, leading to cessation of oxygen flow and potential hypoxaemia.^43^ The entire team must be vigilant for its occurrence, since the pitch change accompanying flow diversion may not be easily audible in busy operating theatres.

If oxygen desaturation occurs (from any cause), the surgeon and assisting team must be adept in swiftly removing surgical instruments to facilitate urgent access for the initiation of rescue oxygenation intervention(s).

### Risk of airway fire

Considering the classical fire triad, where the essential components for fire are a fuel, an ignition source and oxygen,^59^ there has been ongoing international debate regarding the safety of LASER in combination with HFNO and the risk of an airway fire. It has been argued that even in the presence of high fractional inspired oxygen concentrations, the absence of combustible material such as a tracheal tube or jet ventilation catheter, means there is no fuel to burn and fire will not result.^60^ However, it has also been suggested that smouldering surgical debris falling into the reservoir of oxygen is sufficient for ignition (though ignition may not necessarily lead to a sustained flame).

Early case reports and limited retrospective case series in patients receiving HFNO whilst undergoing LASER surgery for endoscopic upper airway pathology reported no airway fires (despite utilising a fractional inspired oxygen concentration of 1.0).^61–64^ However, Onwochei *et al*. published the first case report of an airway fire with HFNO during airway surgery.^53^ They reported brief ignition of a monopolar diathermy sheath during HFNO-ap in a patient undergoing palatal biopsy due to an arc created between the diathermy tip and the patient's titanium dental implant (the patient came to no harm). Though not involving LASER, this prompted many institutions to avoid HFNO use in the context of LASER airway surgery. Some institutions have continued to utilise HFNO-ap during LASER surgery by employing risk mitigation strategies. These include reducing the fractional inspired concentration of oxygen to 0.3 before LASER use and ceasing delivery of HFNO for a predefined time period to allow the fractional inspired concentration of the pharyngeal oxygen reservoir to fall prior to LASER use.^64,65^ However, temporary cessation of gas flow or utilising a reduced fractional inspired concentration of oxygen is likely to reduce the overall duration of effective apnoeic oxygenation.

Trials have been conducted in animal models simulating LASER use during HFNO delivery in upper airway surgery, where the fractional inspired oxygen concentration, LASER energy/mode of firing and endolaryngeal materials were varied (e.g. dry cotton, plastic).^66,67^ Chang *et al*. demonstrated ignition events that led to self-sustained fires even in the absence of foreign combustible materials, with the native tissue and smoke plume igniting.^66^ Baudouin *et al* found no combustion occurred in the absence of foreign endolaryngeal material, though fire did occur in the presence of dry cotton – even when the fractional inspired concentration of oxygen was reduced to 0.3.^67^ Conversely, Khan *et al*.^54^ undertook a trial involving 172 patients undergoing HFNO-ap for laryngeal LASER surgery, that reported no instances of airway fire, despite a fractional inspired oxygen concentration of 1.0. The authors attributed this to strict adherence of their institution's LASER safety protocol, including specifying LASER settings for power, mode (pulsed) and duration. Nevertheless, two further published case reports of fire during HFNO use (one with monopolar diathermy,^68^ and the other involving a facial fire during LASER testing^69^) have reinforced the significant risks involved.

The current evidence surrounding the relative safety of HFNO-ap and LASER is conflicting, and practices vary internationally. This combination should only be contemplated in specialist centres where a thorough fire-risk assessment has been undertaken and an appropriate mitigation strategy has been implemented.^70^ There is no existing national or international published guidance governing this specific area; however, the prevailing consensus in the UK is that *routine* use of HFNO and LASER is not recommended.^71^

## Conclusions

Since Patel and Nouraei's landmark paper a decade ago, HFNO-ap has become a widely adopted oxygenation technique for patients undergoing endoscopic pharyngolaryngeal surgery. Even without a full understanding of the physiological mechanisms and subsequent questioning of the proposed enhanced rate of CO_2_ clearance, it has been proven to be a safe and effective means of oxygenation for this type of surgery in carefully selected patients, providing unrivalled views and unrestricted access to the surgical field. Nevertheless, it remains an advanced airway management technique that requires judicious patient and procedure selection, meticulous planning and thorough preparation. HFNO-ap should be undertaken by a cohesive, highly skilled multidisciplinary team that has a good appreciation of its limitations, including the requirement for an agreed, patient-specific, immediately deliverable rescue oxygenation strategy.
